# Wuzi-Yanzong-Wan inhibits testicular mitochondrial apoptosis in rats by downregulating TAp73-Mediated P38 MAPK-ADAM17 pathway

**DOI:** 10.3389/fphar.2025.1665356

**Published:** 2025-09-08

**Authors:** Ziao Liu, Xiaohan Ni, Jingya Li, Min Pan, Fengqing Xu, Hongsu Zhao, Li Li, Tongsheng Wang

**Affiliations:** ^1^ Department of Physiology and Pharmacology, Anhui University of Traditional Chinese Medicine, Hefei, China; ^2^ College of pharmacy, Anhui University of Traditional Chinese Medicine, Hefei, China

**Keywords:** TAp73, oligoasthenospermia, Wuzi-Yanzong-Wan, P38 MAPK-ADAM17 pathway, mitochondrial apoptotic pathway

## Abstract

**Objective:**

This study investigates the relationship between TAp73 protein overexpression and sperm quality, and evaluates the prophylactic and therapeutic effects of Wuzi-Yanzong-Wan (WZYZW) on oligoasthenospermia (OA) induced by tripterygium glycosides (TGs) and etoposide in rats. Furthermore, it elucidates the underlying mechanism by analyzing the intervention’s role in downregulating TAp73 protein expression and modulating the P38 MAPK-ADAM17 signaling pathway, ultimately providing empirical evidence to support its clinical application.

**Methods:**

Sprague-Dawley (SD) rats received TGs (40 mg/kg/d for 8 weeks) to induce OA. Intervention groups were treated with either WZYZW (1 or 2 g/kg/d) or Shengjing Capsule (SJJN). Meanwhile, a TAp73-overexpressing Sertoli-germ cell co-culture system was established using etoposide (200 μM for 48 h) and treated with WZYZW-containing serum (2.5%–10%), to explore the mechanisms associated with TAp73.

**Results:**

The *in vivo* experimental results demonstrated that WZYZW treatment significantly ameliorated testicular atrophy in model rats, evidenced by increased testicular volume and elevated testicular/epididymal indices. Simultaneously, WZYZW effectively reversed sperm quality impairment in the model group, manifesting as significantly increased sperm density, enhanced sperm motility, and reduced sperm abnormality rate. Furthermore, WZYZW treatment significantly upregulated serum levels of key sex hormones (e.g., testosterone, follicle-stimulating hormone, luteinizing hormone). Mechanistic investigations revealed that WZYZW markedly suppressed germ cell apoptosis (flow cytometry confirmed a significant decrease in apoptotic cell proportion) and attenuated DNA damage (indicated by significantly reduced γ-H2AX (a DNA damage marker) fluorescence intensity). WZYZW effectively restored impaired mitochondrial function and suppressed the expression of key proteins in the mitochondrial apoptotic pathway (e.g., decreased Bax/Bcl-2 ratio, reduced Cyt-c release, inhibited activation of Caspase-9 and Caspase-3). *In vivo* experimental results showed that WZYZW significantly inhibited cell apoptosis, effectively suppressed membrane potential depolarization in TAp73-overexpressing model cells, inhibited the opening of the mitochondrial permeability transition pore, and concurrently suppressed the expression of proteins associated with the mitochondrial apoptotic pathway. Collectively, these changes blocked the TAp73-p38 MAPK/ADAM17 axis-activated mitochondrial apoptotic cascade, thereby exerting its testicular protective effects.

**Conclusion:**

This study highlights that WZYZW effectively alleviates testicular DNA damage and mitochondrial apoptosis, suggesting that its mechanism may be associated with reduced TAp73 expression. These experimental findings provide a novel therapeutic target for the clinical application of WZYZW in OA treatment.

## GRAPHICAL ABSTRACT

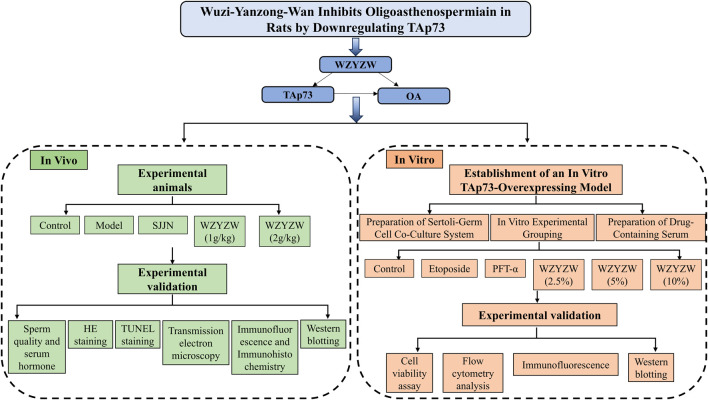

## 1 Introduction

Infertility represents a significant global health challenge, affecting approximately 15% of reproductive-aged couples, with male factors contributing to 50% of cases (Jiao et al., 2021). Among the various causes of male infertility, OA accounts for 21%–51% of cases, establishing it as the most prevalent etiology (Khatun et al., 2018; Schilit, 2019). Given that the pathogenesis of OA remains incompletely understood and effective treatments are limited, it has emerged as a common yet refractory condition impairing male reproductive health. Consequently, elucidating its pathogenic mechanisms and identifying effective therapeutic strategies constitute major research focuses in reproductive medicine (Shan et al., 2020).

TAp73, a member of the p53 tumor suppressor family transcribed from the P1 promoter, induces cell cycle arrest and apoptosis. While extensively studied in tumorigenesis and cancer progression (Ramos et al., 2020), emerging evidence indicates that TAp73 plays a distinct role in regulating sperm quantity and quality. It serves as a critical factor ensuring sperm maturation and fertility, with its dysregulation closely linked to OA pathogenesis (Bozdemir et al., 2025; Gebel et al., 2017; Nemajerova and Moll, 2019). TAp73-knockout mice exhibit reduced sperm density, impaired motility, decreased testicular weight, and increased germ cell apoptosis (Liu et al., 2021). Conversely, TAp73 underexpression induces OA-like phenotypes, while its upregulation prevents cell junction defects in Sertoli-germ cell co-culture systems and protects against premature sperm apoptosis (Li et al., 2024; Holembowski et al., 2014). As a key transcriptional regulator of germ cell apoptosis, TAp73 overexpression activates both the mitochondrial and death receptor apoptotic pathways, triggering excessive germ cell apoptosis through multiple mechanisms (Ito et al., 2024; Moreno et al., 2011). Intratesticular injection of etoposide in rats activates TAp73 in a dose-dependent manner, elevating expression of c-Abl, TAp73, cleaved caspase-3, and BAX proteins, ultimately inducing apoptotic responses in the reproductive system via the c-Abl/TAp73 pathway (Codelia et al., 2010). Thus, TAp73 homeostasis critically influences spermatogenesis and sperm maturation (Xie et al., 2016; Inoue et al., 2014), garnering increasing attention in male reproductive research.

ADAM17, also designated tumor necrosis factor-α converting enzyme (TACE), belongs to the disintegrin and metalloproteinase family. It regulates diverse biological processes including cell adhesion, migration, proteolysis, and signal transduction, primarily by controlling the ectodomain shedding of various receptors (Edwards et al., 2008). Studies report upregulated ADAM17 mRNA and protein levels during etoposide-induced apoptosis in GC-1 and GC-2 spermatogonial cell lines, suggesting its regulatory role in germ cell apoptosis (White et al., 2005). Upon exposure to oxidative stress or DNA damage, c-Abl activates p38 MAPK and PKC, leading to phosphorylation and subsequent activation of ADAM17. This cascade induces shedding of the extracellular domain of the c-kit receptor, disrupting its binding to SCF and ultimately triggering germ cell apoptosis (Böhm et al., 2005; Blobel, 2005). Notably, c-Abl serves as a physiological activator of TAp73, directly targeting and modulating its transcriptional activity to initiate pro-apoptotic signaling. Thus, activation of the P38 MAPK-ADAM17 pathway represents a critical mechanism for inducing germ cell apoptosis.

Tripterygium glycosides (TGs), a lipophilic mixture extracted from the roots of Tripterygium wilfordii, are clinically employed for treating certain autoimmune disorders. However, TGs induce sperm DNA damage, and prolonged administration causes testicular atrophy along with degeneration and necrosis of spermatogenic cells and spermatozoa, resulting in OA (Huovila et al., 2005). A meta-analysis report on the incidence of reproductive toxicity in TGs users revealed that approximately 20.3% of male patients experienced reduced sperm motility. The analysis further indicated that the toxic effects of TGs on the reproductive system primarily manifest in the following aspects: alterations in reproductive tissue structure, damage to germ cells, and disturbances in reproductive hormone levels (Chen et al., 2023). WZYZW is a classic formula for tonifying the kidney and benefiting the essence, which is composed of dodder, wolfberry, raspberry, Schisandra chinensis, and Plantago ovata, and is known as “the first formula of seeds in the ancient and modern world”, and in the early stage, we found that WZYZW had significant efficacy on TGs-induced OA, and it could block spermatozoa mitochondrial apoptotic pathway. its mechanism of repairing sperm DNA damage is not clear (Zhang et al., 2022). Shengjing Capsule (SJJN) was selected as the investigational positive medicine for male infertility treatment in the present study. Both SJJN and WZYZW are approved by the National Medical Products Administration (NMPA) of China for clinical management of male infertility, with their therapeutic mechanisms involving modulation of the hypothalamic-pituitary-gonadal (HPG) axis function and elevation of sex hormone levels (Qu, 2025). Building on the pivotal role of TAp73 in sperm DNA damage repair (Zhou et al., 2023; Mikulenkova et al., 2015), this study employed TGs to establish a rat model with TAp73 overexpression *in vivo*. Concurrently, an *in vitro* TAp73-overexpressing Sertoli-germ cell co-culture system was generated using etoposide, a chemotherapeutic agent commonly used for testicular cancer (Codelia et al., 2010). We investigated the potential mechanism of WZYZW through the lens of modulating the P38 MAPK-ADAM17 pathway. This research offers novel insights into the molecular mechanisms underlying OA pathogenesis and identifies promising therapeutic targets. Furthermore, it provides substantial support for validating the efficacy of WZYZW in treating OA.

## 2 Materials and methods

### 2.1 Materials and reagents

TGs was purchased from Fudan Fuhua (Shanghai, China) and prepared by dissolving in purified water. WZYZW was purchased from Tong Ren tang (Beijing, China) and prepared by dissolving in purified water. Etoposide and Pifithrin-α were purchased from Letai Mei (CAS: 33,419-42-0, Chengdu, China) and Abmole (Cat#: M2036, United States), respectively, and dissolved in DMSO for later use. SJJN was purchased from Liaoyuan Hetang (Guizhou, China) and prepared by dissolving in purified water. The enzyme-linked immunosorbent assay (ELISA) kits for testosterone (T)、Luteinizing Hormone (LH) and follicle-stimulating hormone (FSH) were acquired from Meimian (Jiangsu, China). Antibodies against Cleaved-caspase3 (#99661T) was purchased from Cell Signaling Technology (MA, United States). Antibodies ATM (#R382214), P38/MAPK (#R25239) and ADAM17 (#R381683) were purchased from Zhengneng Biotechnology (Chengdu, China). Antibodies Bcl-2 (#WL01556) and Cyt-c (WL02410) were purchased from Wanlei Biotechnology (Shenyang, China). Antibodies against TAp73 (#66990-1-lg), c-Kit (#18696-1-AP), SCF(#26582-1-AP), BAX (#50599-2-lg) and ADAM17 (#R381683) were purchased from Sanying Biotechnology (Wuhan, China). The secondary antibodies, goat anti-rabbit IgG (#511203) and goat anti-mouse IgG (#511103) antibody were purchased from Zhengneng Biotechnology (Chengdu, China). For immunohistochemistry, antibodies against TAp73 (#WL01604) were purchased from Wanlei Biotechnology (Shenzheng, China).

### 2.2 Experimental animals

50 SPF grade male SD rats (6–8 weeks old, body weight 200–220 g) provided by Jiangsu Huachuang Xinnuo Pharmaceutical Science and Technology Co. Ltd, Certificate of Conformity No.: SCXK (Su) 2020-0009. The rats were placed in an environment with a constant temperature of 25 °C and 50% humidity, and were subjected to a 12/12-h light/dark cycle. They had unrestricted access to food and water. The experimental procedures strictly followed the relevant rules and regulations of the Ethics Committee of Anhui University of Traditional Chinese Medicine (AUTCM), and complied with the university’s animal welfare and ethical review (Ethics No. AHUCM-rats-2024193). After 1 week of acclimatization feeding, the experiment was officially started. (Wu et al., 2021).

Fifty male rats were randomly divided into control group, model group (TGs group, 40 mg/kg/d), positive drug group (SJJN group, 1.6 g/kg/d), and WZYZW low and high dosage groups (1.0 and 2.0 g/kg/d), 10 rats in each group. The rats in all groups except the control group were given TGs suspension (40 mg/kg/d) by gavage for 8 weeks, and the control group was given equal volume of distilled water. Starting from week 5, the rats in each group of administration were treament by gavage at the dose for four consecutive weeks, and the rats in the control and model groups were given an equal volume of distilled water.

### 2.3 LC-MS analysis

The quality of WZYZW was performed on a ExionLC TRIPLE QUAD 4500TM liquid mass spectrometer (AB SCIEX, United States). WZYZW 2g were dissolved in 50 mL of 70% ethanol for LC-MS analysis. Chromatographic conditions: The chromatographic column was an ACQUITY UPLC HSS T3 column (100 mm × 2.1 mm, 1.8 μm), column temperature: 40 °C, flow rate: 0.3 mL/min, injection volume: 1.0 μL, mobile phase A was acetonitrile, and B was 0.1% formic acid in water, and the gradient elution procedure was as follows: 0–2.0 min, 5.0%-15.0%A, 2.0–8.0 min, 15.0%- 30.0%A, 8.0–10.0 min, 30.0%-50.0%A, 10.0–15.0 min, 50.0%- 90.0%A and run at 90%A for 5 min. Mass spectrometry conditions: electrospray ionisation source (ESI) with simultaneous detection of positive and negative ions, air curtain gas (N2): 35 psi, ion source GS1 (N2): 45 psi, GS2 (N2): 50 psi, temperature inside the source 500 °C, spray voltage +5500 V, −4500 V, inlet voltage (EP): +10 V, −10 V, outlet voltage (CXP): +13 V, −13 V. The remaining mass spectrometry parameters are shown in Supplementary Table S1. (CXP): +13 V, −13 V.

### 2.4 Determination of testicular and epididymal organ coefficients

After harvesting the testicles and epididymis, the testes and epididymis were weighed on an analytical balance and the organ coefficient was calculated. Organ coefficient (%) = organ weight (g)/body weight (g) x 100%.

### 2.5 Determination of sperm quality

#### 2.5.1 Determination of sperm density and sperm viability

Fresh right epididymal tissue was placed in a saline solution containing 3 mL of saline preheated to 37 °C, chopped, and incubated for 15 min to allow the mature spermatozoa stored in the epididymis to fully free themselves into the saline solution, and at the end of the incubation, sperm suspensions were prepared by filtering out the tissue fragments through a 200mesh strainer. A drop of sperm suspension was dipped into a Makler sperm counting plate, and the total number of sperm (n) in any 10 compartments was observed under a 200× optical microscope with a sperm density of n×10^6^/mL. Sperm quality was also graded with the following grading criteria:(A) rapid straight forward movement; (B) slow straight forward movement; (C) *in situ* movement; and (D) inactivity. Sperm viability (%) = (A + B)/total spermatozoa × 100%. (Jiao et al., 2021).

#### 2.5.2 Determination of sperm malformation

Sperm smears were prepared by push piece method by taking 10 μL of sperm suspension and dropping it on one side of the slide and left to dry naturally in air. After drying, the sperm smears were fixed in 95% ethanol for 3 min, dried and hydrated for 1–2 min. It was stained with nuclear stain (#D029-1-1, Nanjing, China) in sperm rapid stain for 1 min, rinsed under running water, then stained with plasmodial stain for 1 min and finally rinsed with colour enhancing solution and filter paper to absorb the liquid. After sealing the film with neutral resin, microscopic observation and pictures were taken under microscope oil microscope. The microscopic pictures were subjected to sperm malformation rate counting, counting the number of defective spermatozoa in 200 spermatozoa. Sperm deformity rate (%) = (number of defective spermatozoa/total number of spermatozoa) × 100%.

### 2.6 Determination of hormones

Serum was used to measure sex hormone concentrations. Determination of T, LH and FSH in rat serum using ELISA kit method.

### 2.7 H&E

Tissues of testis and epididymis were fixed with 4% paraformaldehyde, the tissues were dehydrated and cleared, embedded in paraffin wax, sliced and deparaffinised at a thickness of 5 μm by a slicing machine, stained with hematoxylin and eosin staining sequentially, and the tissue slices were scanned and observed under slide scanner (#WS-10, WISLEAP, China) and photographed, and pathological changes in each group were analysed. (Ma et al., 2021).

### 2.8 Preparation of sertoli-germ cell Co-Culture system

Testes were excised from rats and rinsed with pre-cooled D-Hank’s solution. After removing the tunica albuginea, seminiferous tubules were minced in D-Hank’s solution containing antibiotics and antimycotics. The minced tubules were enzymatically digested at 37 °C for 30 min using 1 mg/mL collagenase IV and 0.25% trypsin in the presence of DNase I. Fetal bovine serum (FBS) was added to terminate digestion, and the cell suspension was filtered through a sterile 70-μm nylon mesh. Cells were pelleted by centrifugation at 1,000 rpm for 5 min. The harvested cells were subsequently cultured in DMEM/F12 medium supplemented with 10% FBS and 1% penicillin-streptomycin at 37 °C under 5.0% CO_2_ (Wegner et al., 2013).

### 2.9 Preparation of drug-containing serum

Twenty male Sprague-Dawley (SD) rats (SPF-grade, 6–8 weeks old, 200–220 g) were housed under controlled environmental conditions (25 °C, 50% humidity, 12-h light/dark cycle) with *ad libitum* access to food and water. Rats were randomly assigned to either the WZYZW group (n = 10), receiving 4 g/kg/d Wuzi-Yanzong-Wan (WZYZW) via oral gavage for seven consecutive days, or the control group (n = 10), administered an equivalent volume of saline. One hour following the final administration, blood was collected from the abdominal aorta under anesthesia. Serum samples were obtained by centrifugation at 3,000 rpm for 15 min, filtered through 0.22-μm cellulose acetate membranes, heat-inactivated at 56 °C for 30 min, aliquoted, and cryopreserved at −80 °C for subsequent experimental use. (Wu et al., 2022). The drug-containing serum represents the blood drug concentration level in the dosed animals, and we define this as undiluted drug-containing serum (100% concentration). In subsequent *in vitro* cell experiments, this undiluted serum is diluted into the cell culture medium at specific volume percentages (v/v) to achieve the desired treatment concentrations before being applied to the target cells.

### 2.10 Cell viability assay

Cell viability in the Sertoli-germ cell co-culture system was assessed using the CCK-8 colorimetric assay. Cells (3,000/well) were seeded in 96-well plates and cultured until complete adhesion was achieved. Following stimulation with PFT-α, etoposide (ETO), or drug-containing serum, the culture medium was removed. Subsequently, 100 μL of fresh medium containing 10 μL CCK-8 reagent was added to each well, followed by incubation for 2–4 h. Optical density (OD) at 450 nm was measured using a microplate reader.

### 2.11 Determination of apoptosis in germ cells

#### 2.11.1 Flow cytometry analysis

Take sperm suspension or cell suspension and centrifuge at 1800 rpm for 10 min. Discard the supernatant, add 1 mL of pre-cooled PBS and wash the sample by shaking gently. Centrifuge the sample at 1800 rpm for 10 min, discard the supernatant, and suspend the sample by adding 400 μL of Annexin V affix. Add 5 μL Annexin V-FITC staining solution, mix gently, and incubate at 2 °C–8 °C for 15 min away from light. Add 10 μL PI staining solution, mix and incubate for 5 min at 2 °C–8 °C away from light. Cell apoptosis rate was detected by flow cytometry. Dead cell in the Q1 quadrant, apoptotic cell in the Q2 and Q4 quadrants, and normal cell in the Q3 quadrant. Cell apoptosis rate (%) = (proportion of Q2 quadrant + proportion of Q4 quadrant) × 100%.

#### 2.11.2 TUNEL analysis

Testicular tissue wax blocks were sectioned, deparaffinised, dehydrated, and incubated dropwise with proteinase K solution (20 μg/mL) for 20 min at room temperature. At the end of incubation, endogenous catalase inhibitor was added dropwise to block the endogenous reduction reaction. Then biomarkerin solution was added dropwise, and incubated at 37 °C for 1 h, avoiding light, then Streptavidin-HRP working solution was added dropwise, and incubated at room temperature, avoiding light, for 30 min, and then DAB chromogenic solution was added dropwise to make the broken DNA double strands colourful. Finally, the nuclei were stained with hematoxylin, so that the stained broken DNA double strands were distinguished from the normal hematoxylin-stained nuclei. At the end of the staining, the slides were sealed with neutral resin, and the stained slides were scanned by a slide scanner, photographed and analysed, in which the nuclei of the cells with broken DNA double strands would appear yellowish-brown as a positive result, and the blue-purple nuclei would be a negative result, and the apoptosis rate of the cells was calculated by selecting 200 germ cells. Germ cell apoptosis rate (%) = (number of positive cells/total number of cells) ×100%.

### 2.12 Immunofluorescence staining

Both tissue sections and cell culture plates were covered with fixative for 5–15 min. After fixation, blocking buffer was applied and incubated at room temperature for 15 min. Following blocking buffer removal, rabbit anti-γ-H2AX primary antibody was added and incubated overnight at 4 °C. The primary antibody was then aspirated, and Cy3-conjugated anti-rabbit secondary antibody was applied for 1-h incubation in darkness. Nuclei were counterstained with DAPI. Finally, samples were mounted with antifade mounting medium and coverslipped. Fluorescent images were captured using an inverted fluorescence microscope. γ-H2AX expression levels were quantified by measuring red fluorescence intensity with ImageJ software.

### 2.13 Western blot analysis

Testicular tissues and co-cultured cells were lysed in RIPA buffer containing protease inhibitors. Lysates were centrifuged at 12,000 × g for 15 min at 4 °C. Supernatants were collected and stored at −80 °C for subsequent analysis. Protein extracts were mixed with 5× loading buffer and denatured at 95 °C for 5 min. Proteins were separated on 8% SDS-polyacrylamide gels through electrophoresis at 80 V (stacking gel) and 120 V (separating gel). Following electrophoretic separation, proteins were transferred onto 0.45-μm PVDF membranes at 200 mA for 2 h at 4 °C. Membranes were blocked with 5% non-fat milk for 2 h at room temperature, then incubated overnight at 4 °C with primary antibodies diluted in 5% BSA. After washing, membranes were probed with horseradish peroxidase (HRP)-conjugated rabbit secondary antibodies for 1 h. Protein bands were visualized using ECL chemiluminescence detection. Band intensity was quantified with ImageJ software to determine protein expression levels.

### 2.14 Mitochondrial function assessments

#### 2.14.1 Mitochondrial membrane potential (ΔΨm) assay

Testicular tissue (100 mg) was homogenized in 1 mL ice-cold Lysis Buffer from a mitochondrial isolation kit. Crude mitochondria were extracted via differential centrifugation, washed with 0.5 mL Wash Buffer, and pelleted by repeated centrifugation. Purified mitochondrial pellets were resuspended in 100 μL Store Buffer. JC-1 working solution was added to purified mitochondria/cell cultures and incubated for 10 min. Fluorescent images were captured using an inverted fluorescence microscope and analyzed with ImageJ. At high ΔΨm, JC-1 forms J-aggregates emitting red fluorescence (590 nm); at low ΔΨm, it remains monomeric emitting green fluorescence (530 nm). The red/green fluorescence ratio quantifies mitochondrial depolarization: decreased ratios indicate progressive ΔΨm loss and mitochondrial impairment.

#### 2.14.2 Testicular ultrastructural evaluation

Testes fixed in 2.5% glutaraldehyde were dehydrated through ethanol gradients, infiltrated with propylene oxide:epoxy resin (1:1) for 2 h, and embedded in pure epoxy resin. Polymerized blocks were sectioned at 70 nm thickness. Ultrathin sections were double-stained with uranyl acetate and lead citrate, then examined under transmission electron microscopy (TEM) to document ultrastructural alterations. (Tassis et al., 2020).

#### 2.14.3 Mitochondrial permeability transition pore (MPTP) opening assay

After PBS washing, cells were incubated with Calcein AM staining solution and CoCl_2_-based fluorescence quencher working solution (to quench cytosolic signal) for 45–70 min at 37 °C in darkness. The solution was replaced with pre-warmed medium and incubated for 30 min. Following PBS washes (×3), cells were imaged in assay buffer using fluorescence microscopy. Mitochondrial Calcein fluorescence intensity (ex/em: 488/515 nm) inversely correlates with MPTP opening: higher green intensity indicates reduced MPTP opening and lesser mitochondrial damage, while diminished fluorescence reflects increased MPTP activity and severe injury. ImageJ quantified fluorescence intensities.

### 2.15 Immunohistochemistry

After deparaffinisation and dehydration of paraffin testis tissue sections, the sections were boiled in sodium citrate buffer for 15 min to repair the antigens and then endogenous peroxidase blocker was added dropwise. Afterwards, the sections were incubated with 5% bovine serum albumin (BSA) for 15 min at room temperature for sealing. In the next step, TAp73 (IHC, 1:200, purchased from Wanleibio, ShenYang, China) primary antibody was added dropwise, so that the primary antibody binds to the specific site on the tissue, and then horseradish peroxidase-labelled HRP secondary antibody was added dropwise, and the colour was developed by the action of DAB chromogenic solution. Nuclear were stained with hematoxylin, and at the end of staining the slides were blocked with neutral resin, and the immunohistochemistry slides were scanned with a slide scanner and photographed for analysis. The blue-purple colour is the nucleus of germ cells, and the yellow-brown colour is the positive result, which represents the distribution and expression of TAp73 protein.

### 2.16 Statistical analysis

The experimental data were analyzed and processed using SPSS 27.0. Normality of the data was confirmed prior to analysis. Mean comparisons were performed using one-way ANOVA. When the ANOVA indicated statistically significant differences, *post hoc* analyses were conducted: Bonferroni’s test was applied under the assumption of homogeneity of variance, whereas Games-Howell’s test was employed for heterogeneous variances, with statistical significance defined at *P* < 0.05.

## 3 Results

### 3.1 WZYZW ameliorates oligoasthenospermia in rats

**FIGURE 1:**
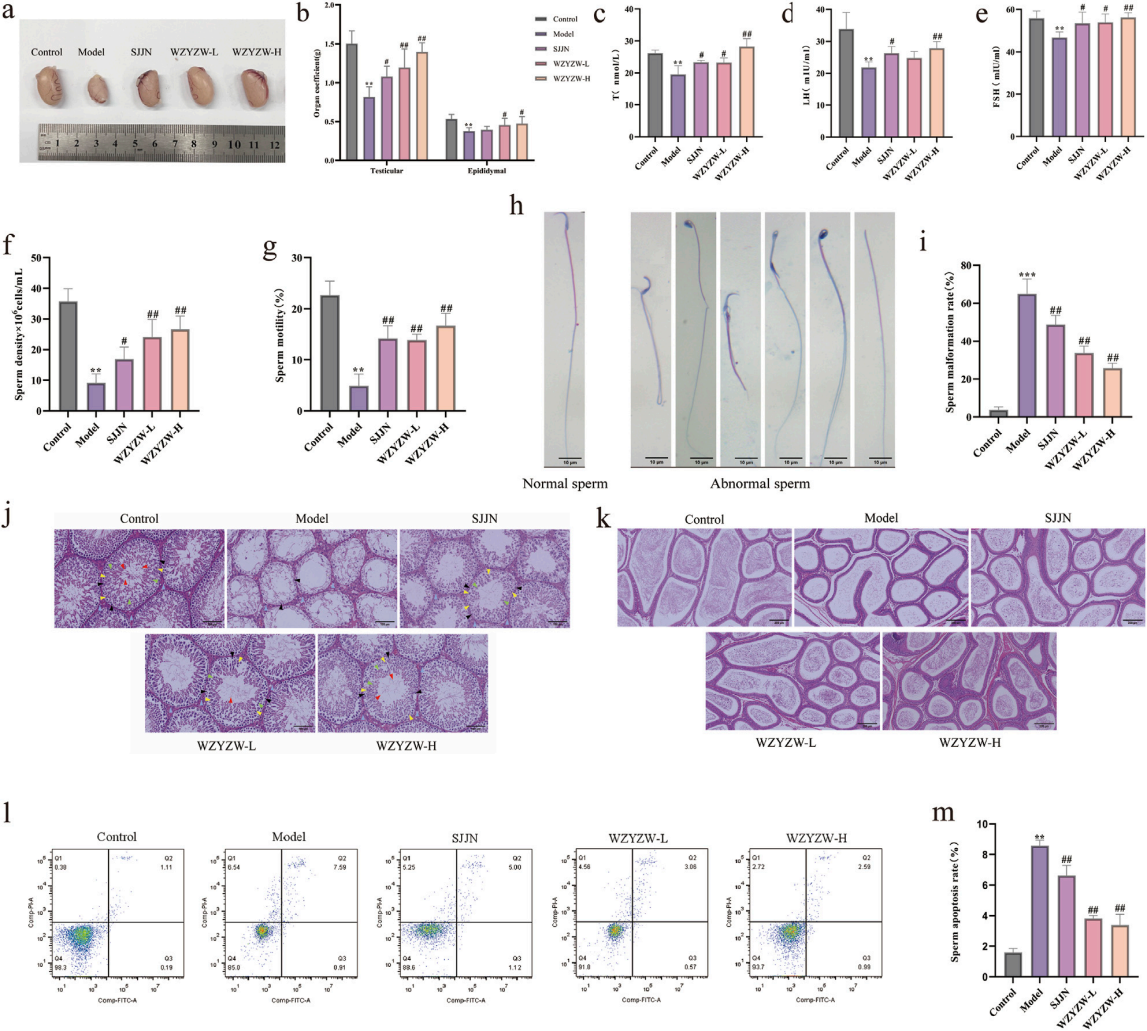
WZYZW ameliorates OA in rats (mean ± SD): **(a)** Testicular morphology; n = 8 **(b)** Testicular/epididymal indices; n = 8 **(c–e)** Serum sex hormones (T, LH, FSH); n = 6 **(f,g)** Sperm density and motility; n = 8 **(h,i)** Sperm abnormality rate; n = 3 **(j)** Testicular histopathology (HE staining, ×200). The diagram uses triangular markers to label several types of cells: blue indicates Leydig cells, black represents spermatogonia, yellow denotes primary spermatocytes, green marks secondary spermatocytes, and red is spermatozoa; n = 3 **(k)** Epididymal histopathology (HE staining, ×200); n = 3 **(l,m)** Sperm apoptosis rate. n = 3. ^*^ *P* < 0.05, ^**^ *P* < 0.01, ^***^ *P* < 0.001 vs. control group; ^#^ *P* < 0.05, ^##^ *P* < 0.01 vs. model group.

TGs-induced model rats exhibited significant testicular atrophy with markedly reduced volume (Figure 1a) and significantly decreased testicular/epididymal indices (*P* < 0.01). Pharmacological interventions restored near-normal testicular size and improved organ coefficients across treatment groups, with the high-dose WZYZW (2 g/kg) group demonstrating the most substantial recovery (*P* < 0.01, Figure 1b). Serological analysis revealed significantly suppressed serum T, LH, and FSH levels in model rats (*P* < 0.01), which were effectively elevated by both SJJN and WZYZW treatments, particularly at the 2 g/kg WZYZW dose (*P* < 0.01). Sperm parameter analysis showed severely compromised sperm density and motility (*P* < 0.01, Figures 1f,g) alongside significantly increased abnormal sperm morphology—including detached tails, bent necks, swollen heads, headless sperm, and coiled forms (*P* < 0.001, Figures 1h,i)—all of which were significantly ameliorated post-treatment, with improved density/motility (*P* < 0.01) and reduced abnormality rates. Histopathological examination revealed severe seminiferous tubule damage in model testes: tubule shrinkage, reduced diameter, disorganized spermatogenic epithelium with diminished layers, prominent vacuolization, and absence of developing germ cells (Figure 1j); epididymal sections showed luminal sperm depletion, widened ducts, disordered arrangement, and principal cell vacuolization (Figure 1k). Conversely, treated groups—especially WZYZW—displayed attenuated pathology: improved tubule structure with increased diameter, multilayered/organized spermatogenic epithelium containing developing cells, normalized epididymal ducts with thickened walls, aligned stereocilia, and repopulated sperm. Flow cytometry confirmed significantly elevated sperm apoptosis in model group (*P* < 0.01), which was effectively reduced across treatment groups, with optimal efficacy in the 2 g/kg WZYZW group. Collectively, these findings demonstrate WZYZW’s significant protection against TGs-induced testicular injury through restoration of spermatogenic function and hormonal homeostasis. Ten active ingredients were detected during the quality control of WZYZW (Supplementary Table S1), including betaine 0.568 mg/g, hyperoside 0.722 mg/g, schizandrin 0.0239 mg/g, deoxyschizandrin 0.0544 mg/g, ellagic acid 0.0754 mg/g, geniposidic acid 0.303 mg/g, acteoside 0.261 mg/g, kaempferol 3-rutinoside 0.0188 mg/g, quercetin 0.0547 mg/g, kaempferol 0.0312 mg/g. The total ion chromatogram see (Supplementary Figure S1).

### 3.2 WZYZW inhibits testicular apoptosis

**FIGURE 2:**
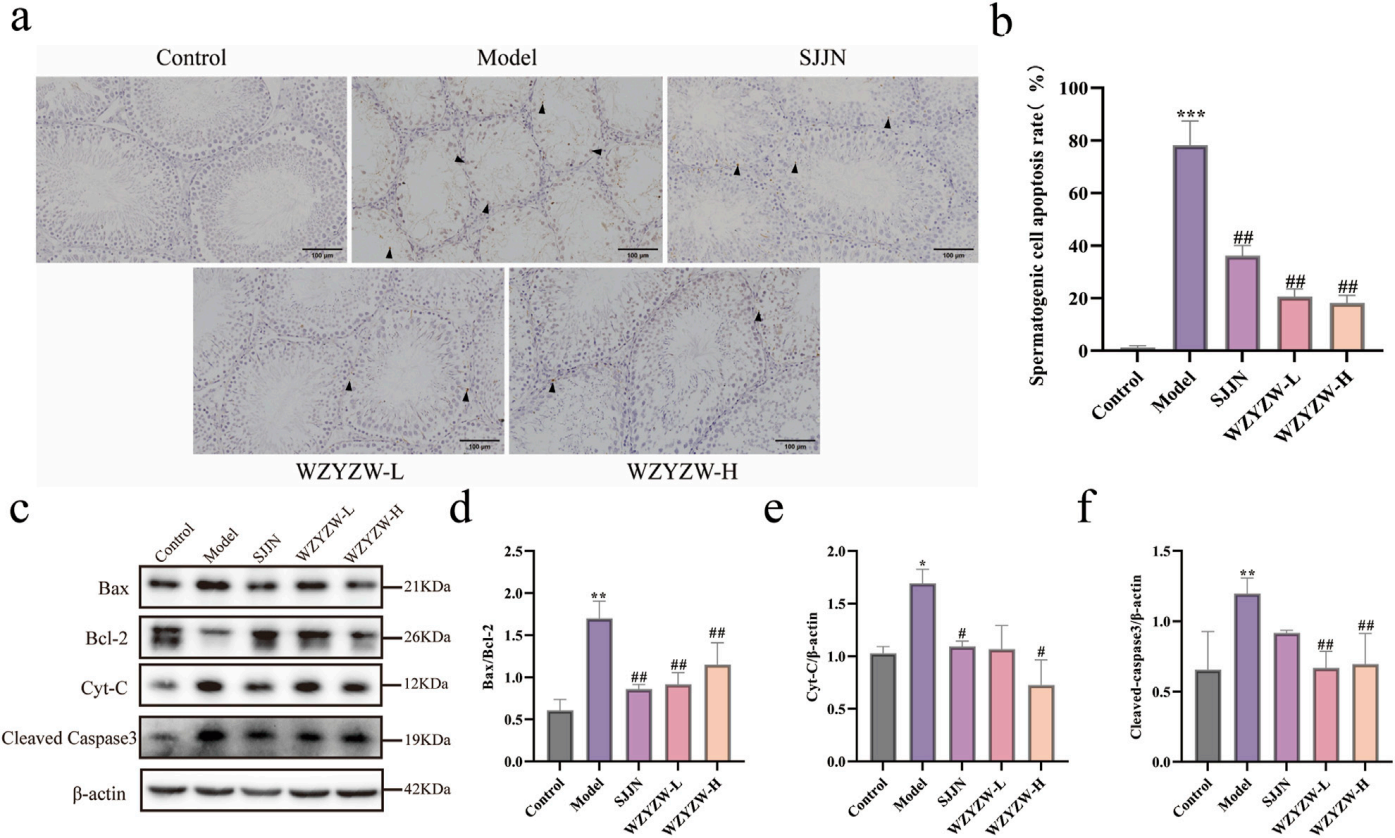
WZYZW suppresses testicular apoptosis in rats (mean ± SD): **(a,b)** TUNEL staining of testicular sections showing apoptotic cells, the figure uses black triangular markers to label positive cells. (×200 magnification) n = 3; **(c‐f)** Western blot analysis of apoptosis-related proteins (BAX/Bcl-2 ratio, cytochrome c, cleaved caspase-3) n = 3. ^*^ *P* < 0.05, ^**^ *P* < 0.01, ^***^ *P* < 0.001 vs. control group; ^#^ *P* < 0.05, ^##^ *P* < 0.01 vs. model group.

To evaluate the impact of WZYZW on testicular tissue, apoptosis was assessed via TUNEL staining and Western blot analysis. TUNEL staining revealed that the TGs model group exhibited atrophic seminiferous tubules with conspicuous shrinkage, reduced spermatogenic cells, and significantly increased TUNEL-positive cells compared to controls (*P* < 0.01; Figures 2a,b). WZYZW administration alleviated structural damage in seminiferous tubules and markedly reduced apoptotic cells (*P* < 0.01). Western blot analysis of key mitochondrial apoptotic pathway proteins further demonstrated significantly elevated levels of pro-apoptotic indicators in model testes: BAX/Bcl-2 ratio, cytochrome c (Cyt-C), and cleaved caspase-3 (*P <* 0.01). WZYZW intervention effectively reversed these alterations, significantly suppressing BAX/Bcl-2 ratio, Cyt-C, and cleaved caspase-3 expression (*P* < 0.01), collectively indicating its potent anti-apoptotic effects in testicular tissue.

### 3.3 WZYZW mitigates testicular mitochondrial damage

**FIGURE 3:**
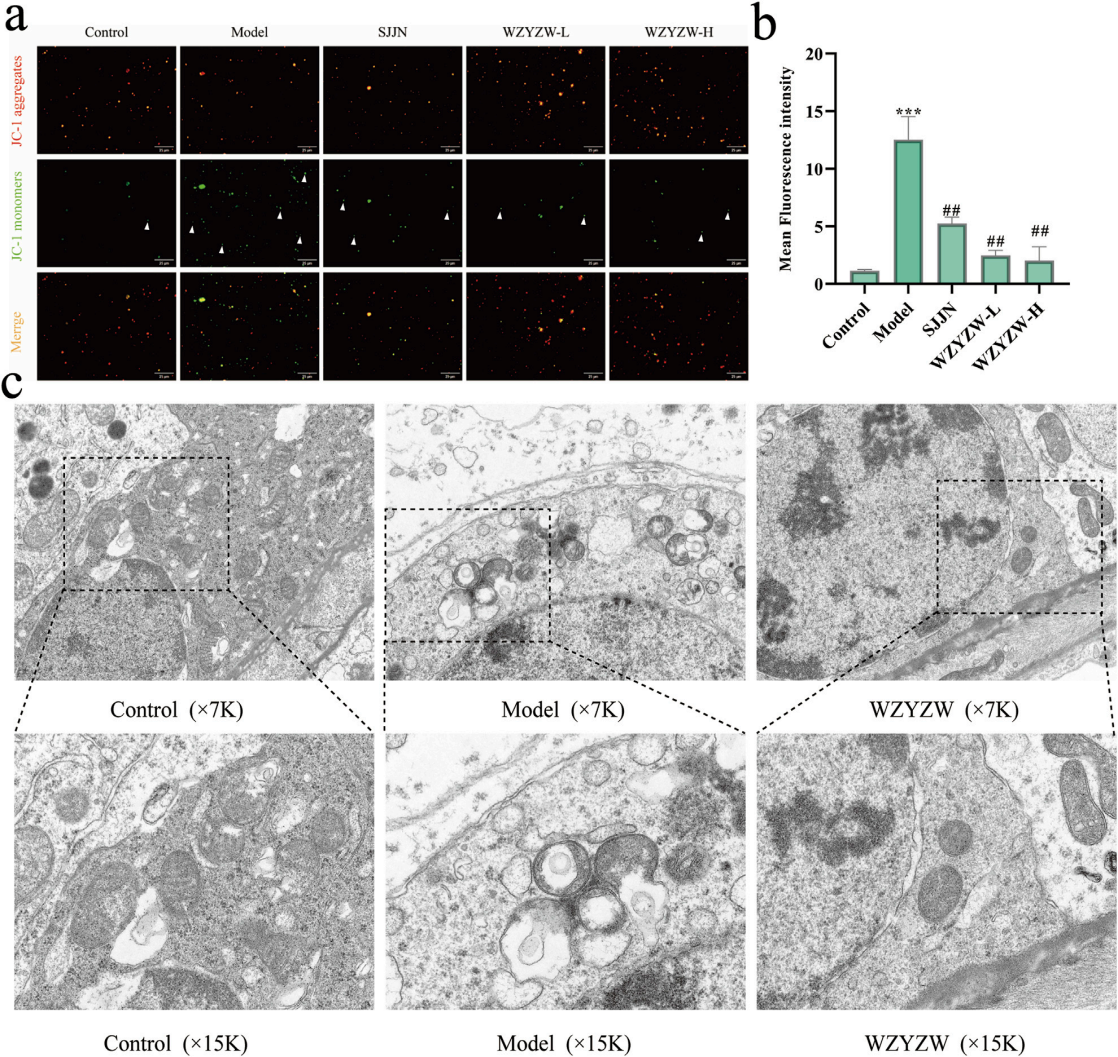
WZYZW attenuates testicular mitochondrial damage in rats (mean ± SD): **(a,b)** JC-1 assay for mitochondrial membrane potential (ΔΨm), the figure employs white triangular markers to indicate cells exhibiting green fluorescence from monomeric JC-1 in isolated mitochondria, a signature of mitochondrial membrane potential depolarization. (×200 magnification) n = 3; **(c)** TEM of testicular ultrastructure n = 3. ^**^ *P* < 0.01 vs. control group; ^##^ *P* < 0.01 vs. model group.

To elucidate mechanisms underlying testicular apoptosis, mitochondrial integrity was evaluated (Figure 3). JC-1 assay revealed significantly intensified green fluorescence (monomeric form) in the TGs model group, indicating severe mitochondrial membrane potential (ΔΨm) dissipation *versus* controls (*P* < 0.001). WZYZW treatment dose-dependently attenuated green fluorescence intensity, with the 2 g/kg dose effectively restoring ΔΨm (*P* < 0.01). Transmission electron microscopy demonstrated control spermatocytes contained abundant mitochondria with organized alignment, intact double membranes, and distinct cristae. Conversely, the model group exhibited markedly reduced mitochondrial numbers, prevalent swelling, ruptured membranes, cristae disintegration, and prominent vacuolization. WZYZW intervention significantly restored mitochondrial density, minimized swelling, preserved membrane integrity, partially visible cristae, and eliminated vacuolization, collectively indicating structural recovery.

### 3.4 WZYZW suppresses the TAp73-P38 MAPK-ADAM17 pathway in testicular tissue

**FIGURE 4:**
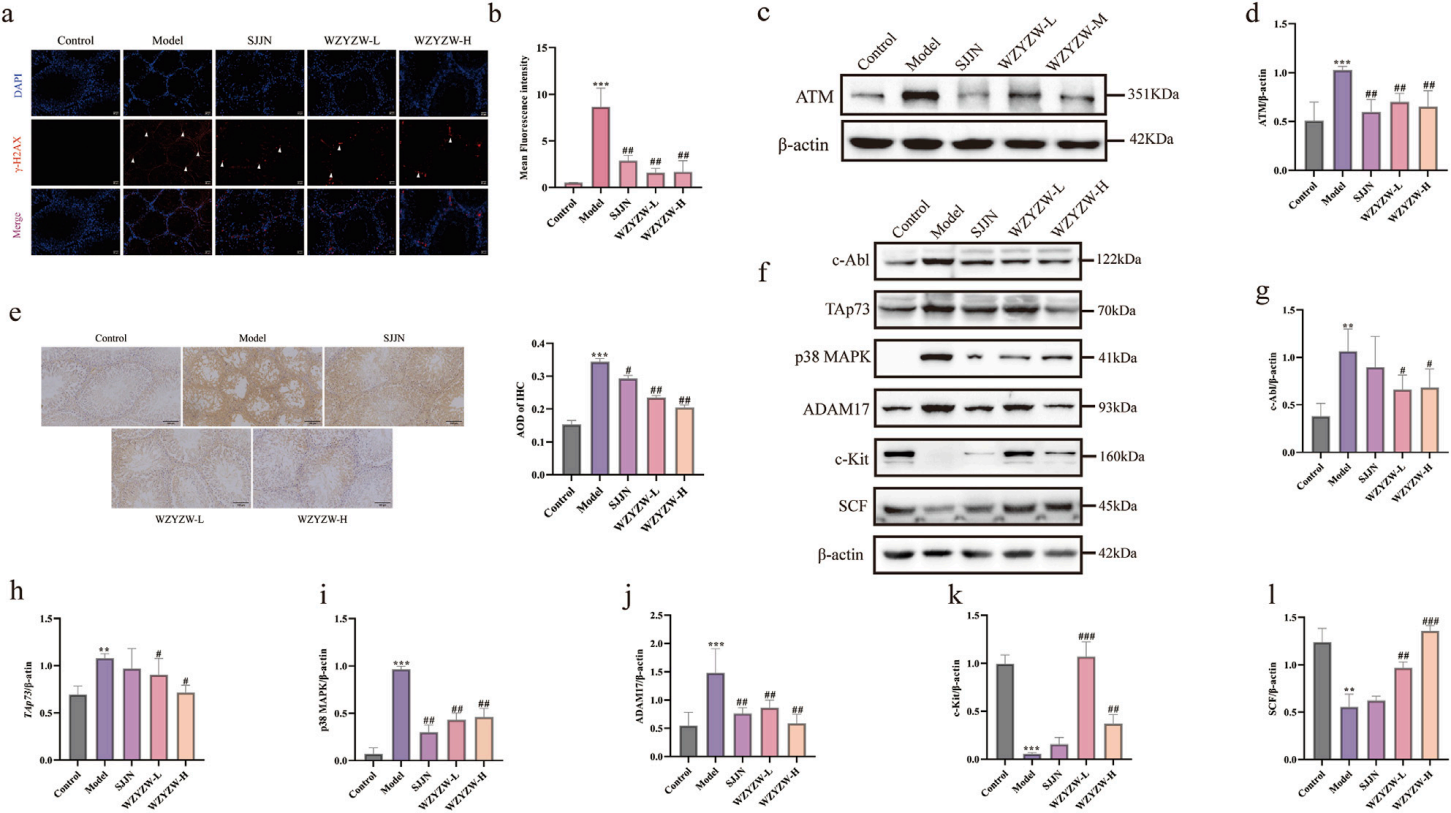
WZYZW suppresses the TAp73-P38 MAPK-ADAM17 pathway in rat testes (mean ± SD): **(a, b)** γ-H2AX IF staining in testicular sections (×200 magnification), white triangular symbols denote γH2AX-positive domains in testicular sections, reflecting quantitative expression and spatial distribution patterns of this histone variant; n = 3; **(c,d)** ATM protein expression analysis n = 3; **(e)** Immunohistochemical detection of TAp73 in spermatogenic cells n = 3; **(f–l)** Expression levels of P38 MAPK-ADAM17 pathway-related proteins n = 3. ^**^ *P* < 0.01, ^***^ *P* < 0.001 vs. control group; ^#^ *P* < 0.05, ^##^ *P* < 0.01, ^###^ *P* < 0.001 vs. model group.

To investigate TGs-induced DNA damage and therapeutic intervention, γ-H2AX IF staining revealed significantly elevated fluorescence intensity and positive cell counts in model testes *versus* controls (*P* < 0.01), indicating severe germ cell DNA damage (Figures 4a,b). WZYZW treatment significantly reduced γ-H2AX signals across all doses (*P* < 0.05), demonstrating effective DNA damage mitigation. Consistently, ATM—a key DNA damage response protein—was significantly upregulated in model group (*P* < 0.001), while WZYZW suppressed its overexpression (*P* < 0.05). Immunohistochemistry (Figure 4e) showed increased TAp73-positive spermatogenic cells in model seminiferous tubules (*P* < 0.05), which was dose-dependently reversed by WZYZW (2 g/kg most effective). Western blot analysis (Figures 4f–l) confirmed significant upregulation of pro-apoptotic/stress proteins (c-Abl, ADAM17, TAp73, p38 MAPK; all *P* < 0.05) in model testes, all significantly downregulated post-WZYZW treatment (*P* < 0.05). Conversely, expression of c-Kit receptor and its ligand SCF—critical for spermatogenic maintenance—was suppressed in model group (*P* < 0.05) with impaired binding, whereas WZYZW restored their expression (*P* < 0.05). Collectively, WZYZW (2 g/kg) antagonizes TGs-induced DNA damage, promotes c-Kit/SCF pathway restoration, and suppresses aberrant TAp73-P38 MAPK-ADAM17 activation.

### 3.5 WZYZW suppresses the TAp73-P38 MAPK-ADAM17 pathway in Co-Cultured cells

**FIGURE 5:**
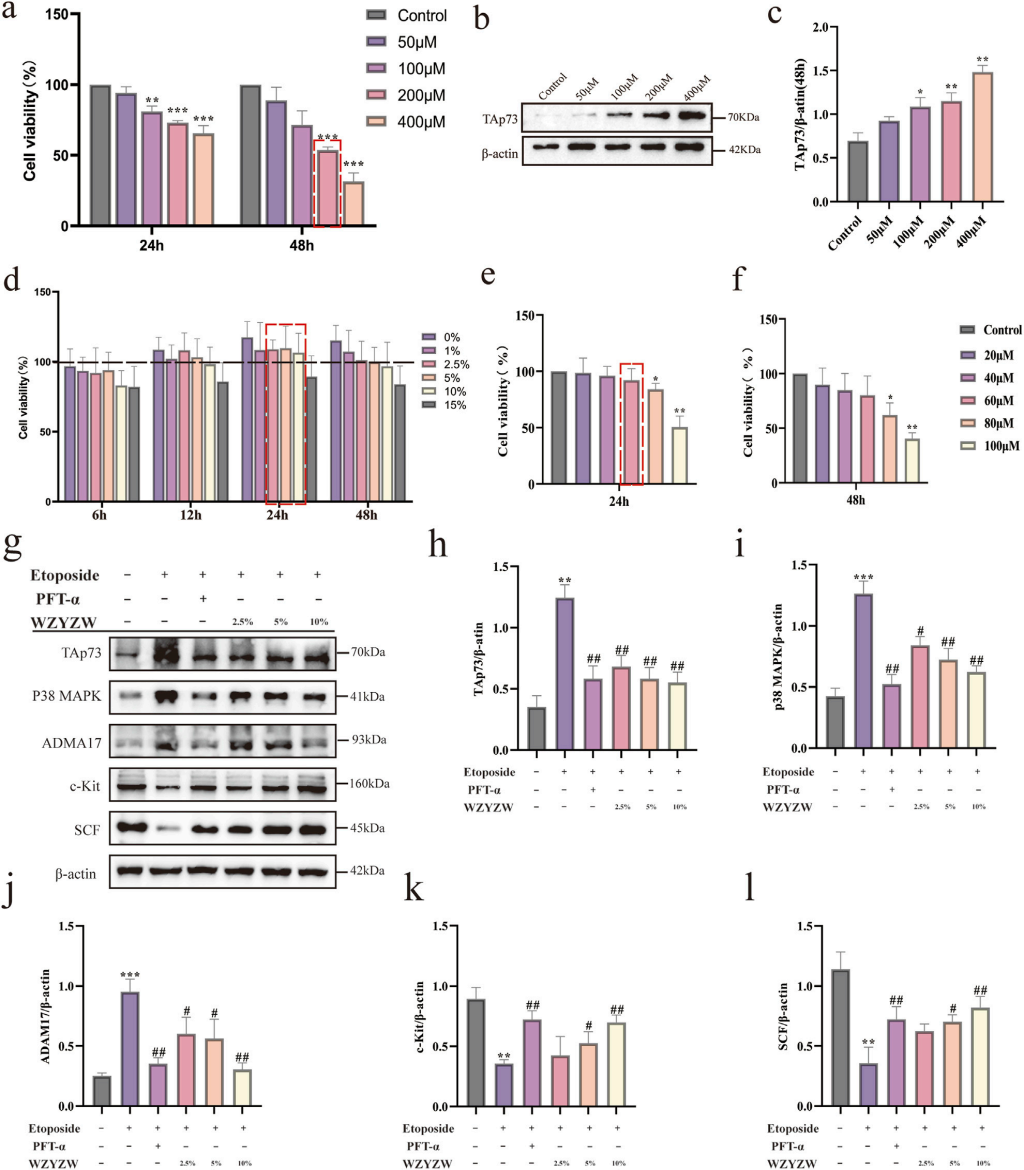
WZYZW suppresses the TAp73-P38 MAPK-ADAM17 pathway in the Sertoli-germ cell co-culture system (mean ± SD): **(a)** Cell viability after 24/48 h etoposide exposure (50–400 μM) n = 3; **(b,c)** TAp73 protein expression post-48 h etoposide treatment n = 3; **(d)** Viability under WZYZW-containing serum (1%–15%, 6–48 h) n = 3; **(e,f)** Viability after PFT-α treatment (24/48 h) n = 3; **(g–l)** Expression of TAp73-P38 MAPK-ADAM17 pathway-related proteins n = 3. ^*^ *P* < 0.05, ^**^ *P* < 0.01, ^***^ *P* < 0.001 vs. control group; ^#^ *P* < 0.05, ^##^ *P* < 0.01 vs. model group.

To establish an *in vitro* germ cell injury model and evaluate WZYZW’s protective effects, CCK-8 assays demonstrated concentration- and time-dependent reductions in cell viability following etoposide (50–400 μM, 24–48 h), with 200 μM etoposide (48 h) achieving about 50% viability (IC_50_; Figure 5a). Western blot confirmed concentration-dependent TAp73 upregulation under these conditions, significant at 200 μM (*P* < 0.05; Figures 5b,c). Subsequent experiments adopted this model (200 μM etoposide, 48 h). Safety assessments revealed WZYZW-containing serum (2.5%–10%, 24 h) caused no significant viability reduction *versus* controls (Figure 5D), while 60 μM PFT-α showed minimal toxicity (Figures 5e,f). Thus, interventions used 2.5%/5%/10% WZYZW serum or 60 μM PFT-α for 24 h. Western blot analysis (Figures 5g–l) revealed etoposide-injured cells exhibited significant upregulation of pro-injury/apoptotic proteins (ADAM17, TAp73, p38 MAPK; *P* < 0.01) and downregulation of spermatogenesis-associated proteins (c-Kit, SCF; *P* < 0.01). Both WZYZW serum and PFT-α significantly suppressed ADAM17/TAp73/p38 MAPK overexpression (*P* < 0.01) while restoring c-Kit/SCF expression (*P* < 0.01). These *in vitro* molecular alterations align with prior *in vivo* findings, validating WZYZW’s mechanistic role in antagonizing germ cell injury via TAp73-p38 MAPK-ADAM17 pathway modulation.

### 3.6 WZYZW attenuates apoptosis in Co-Cultured cells via TAp73 downregulation

**FIGURE 6:**
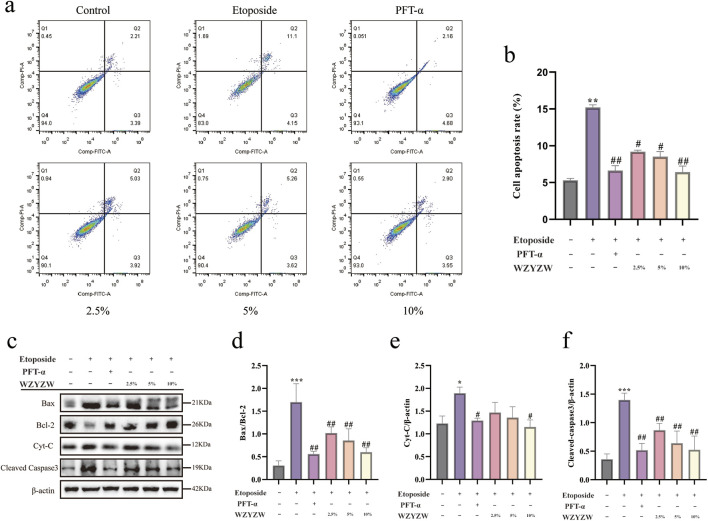
WZYZW mitigates mitochondrial apoptosis in the co-culture system (mean ± SD): **(a,b)** Flow cytometry analysis of apoptosis n = 3; **(c–f)** Western blot detection of apoptosis-related proteins (BAX/Bcl-2 ratio, cytochrome c, cleaved caspase-3) n = 3. ^*^ *P* < 0.05, ^**^ *P* < 0.01, ^***^ *P* < 0.001 vs. control group; ^#^ *P* < 0.05, ^##^ *P* < 0.01 vs. model group.

Apoptosis inhibition by WZYZW was evaluated in the *in vitro* co-culture system. Flow cytometry (Figures 6g,h) confirmed significantly elevated apoptosis rates in etoposide-treated model cells *versus* controls (*P* < 0.01), which were effectively reduced by all drug treatments (*P* < 0.01). Western blot analysis (Figures 6i–l) demonstrated high consistency with *in vivo* findings: Model cells exhibited significantly increased BAX/Bcl-2 ratio, cytochrome c (Cyt-C), and cleaved caspase-3 levels (*P* < 0.01), whereas drug interventions markedly suppressed these pro-apoptotic proteins (*P* < 0.01). These results validate that WZYZW antagonizes mitochondrial apoptosis by downregulating TAp73-mediated P38 MAPK-ADAM17 signaling.

### 3.7 WZYZW attenuates mitochondrial damage via TAp73 downregulation

**FIGURE 7:**
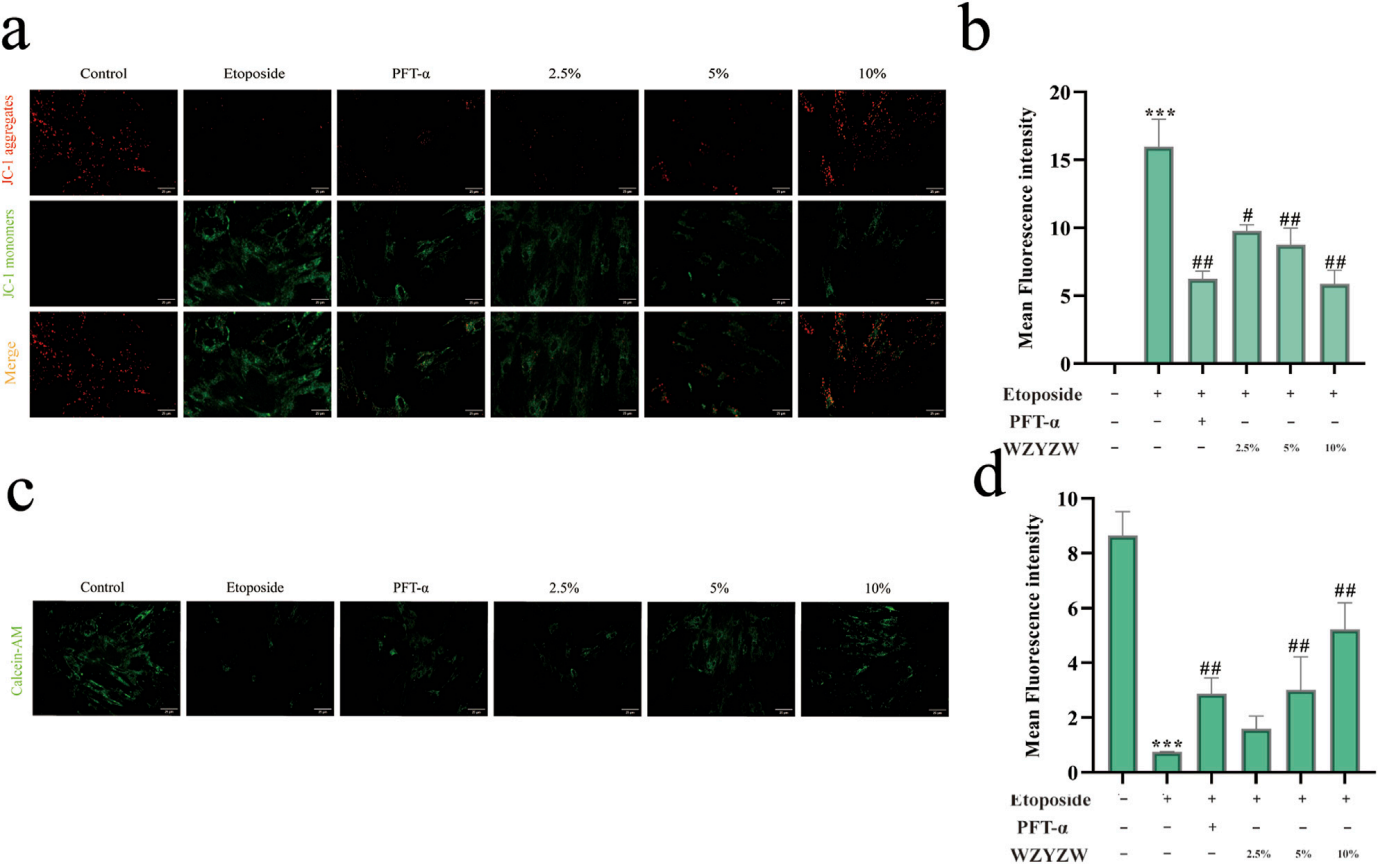
WZYZW attenuates mitochondrial damage (mean ± SD): **(a,b)** JC-1 assay for mitochondrial membrane potential (ΔΨm) in co-cultured cells (×200 magnification) n = 3; **(c)** TEM of testicular ultrastructure n = 3; **(c,d)** MPTP opening assessment in co-culture system (Calcein AM staining, 200×) n = 3. ^*^ *P* < 0.01 vs. control group; ^#^ *P* < 0.05, ^##^ *P* < 0.01 vs. model group.

JC-1 fluorescence staining demonstrated significantly intensified green fluorescence (indicating ΔΨm dissipation) in etoposide-treated model cells *versus* controls. WZYZW intervention substantially reversed this effect, consistent with *in vivo* observations (Figures 7a,b). Further assessment of mitochondrial permeability transition pore (MPTP) opening using Calcein AM/CoCl_2_ quenching revealed that mitochondrial Calcein fluorescence intensity inversely correlates with MPTP activity (higher opening = weaker fluorescence). Model cells exhibited drastically reduced mitochondrial fluorescence *versus* controls (*P* < 0.001), indicating aberrant MPTP opening. WZYZW (particularly 10% serum group) significantly increased fluorescence intensity (*P* < 0.01), effectively suppressing pathological MPTP activation (Figures 7c,d). Collectively, by downregulating TAp73-mediated P38 MAPK-ADAM17 signaling, WZYZW stabilizes ΔΨm, inhibits MPTP overactivation, and preserves ultrastructural integrity, thereby safeguarding mitochondrial function and providing organelle-level mechanistic insights for its anti-apoptotic efficacy.

## 4 Discussion

Spermatogenic cell apoptosis is essential for normal testicular function, but excessive apoptosis frequently leads to OA (Meydanli et al., 2018). γ-H2AX—a phosphorylated product of histone H2AX at serine 139 mediated by ATM in response to DNA double-strand breaks (DSBs)—serves as a quantitative biomarker of DNA damage. While structurally restored DNA permits cell cycle progression, persistent damage triggers apoptosis (Hamer et al., 2001; Talibova et al., 2022). Under pathological conditions (e.g., reproductive infections, chronic inflammation, oxidative stress, ionizing radiation), ATM kinase recognizes DSBs, undergoes autophosphorylation, and activates c-Abl. Activated c-Abl phosphorylates TAp73 at Tyr99, prolonging its half-life and enhancing TAp73-mediated transcriptional activity and pro-apoptotic function. Activated TAp73 further stimulates ADAM17 via the p38 MAPK pathway, which hydrolyzes the c-Kit receptor on germ cells. c-Kit hydrolysis reciprocally amplifies TAp73 expression, ultimately triggering BAX translocation to mitochondrial membranes. This reduces the Bcl-2/BAX ratio, induces MPTP opening, and activates the mitochondrial apoptotic pathway, culminating in pathological germ cell apoptosis.

Ricardo D. Moreno et al. established ADAM17’s regulatory role in germ cell apoptosis, proposing a mechanistic model linking DNA damage to apoptosis (Moreno et al., 2011; Codelia et al., 2010). In irradiated p53 (−/−) mice, despite unchanged TAp73 levels, elevated c-Abl expression interacted with TAp73, inducing spermatogonial apoptosis (Jin et al., 2022). Our study demonstrates that TGs-administered rats exhibit increased testicular DNA damage markers (γ-H2AX), elevated TAp73, reduced sperm motility/density, and heightened abnormality rates—confirming OA pathogenesis. *In vitro*, etoposide upregulated γ-H2AX and TAp73 while increasing apoptosis. Critically, WZYZW reduced DNA damage, suppressed TAp73 overexpression, inhibited the P38 MAPK-ADAM17 pathway, and decreased germ cell apoptosis, thereby preventing OA.

Mitochondrial membrane potential (ΔΨm) and MPTP status are pivotal indicators of mitochondrial health. Significantly reduced ΔΨm is observed in oligozoospermic, asthenozoospermic, and OA patients, directly correlating with sperm count, morphology, and motility (Durairajanayagam et al., 2021). Bcl-2/BAK-mediated mitochondrial permeabilization dissipates ΔΨm, releasing Cyt-C into the cytosol. This sequentially activates caspase-9 and caspase-3, executing mitochondrial apoptosis (Ni et al., 2024). Our *in vivo* and *in vitro* results align: model groups showed ΔΨm dissipation, elevated BAX/Cyt-C/cleaved caspase-3, and pathological MPTP opening—consistent with reported mechanisms of male reproductive injury (Zhang et al., 2022; Simon et al., 2019).

As a clinically established formula for OA (Zhimin et al., 2023), WZYZW significantly downregulated c-Abl, ADAM17, TAp73, and p38 MAPK, confirming suppression of the TAp73-P38 MAPK-ADAM17 axis. The receptor tyrosine kinase c-Kit, upon binding SCF, undergoes autophosphorylation to maintain spermatogonial stem cells and regulate self-renewal. Notably, SCF/c-Kit signaling upstream of Bcl-2/BAX increases Bcl-2 expression when activated (Li et al., 2020). WZYZW elevated c-Kit/SCF expression and the Bcl-2/BAX ratio while reducing Cyt-C/cleaved caspase-3. Concomitantly, it improved sperm parameters, reduced morphological abnormalities, increased serum testosterone, alleviated testicular/epididymal histopathology, and mitigated mitochondrial damage. Collectively, WZYZW counters OA primarily by inhibiting the mitochondrial apoptotic pathway in germ cells.

## 5 Conclusion

**FIGURE 8:**
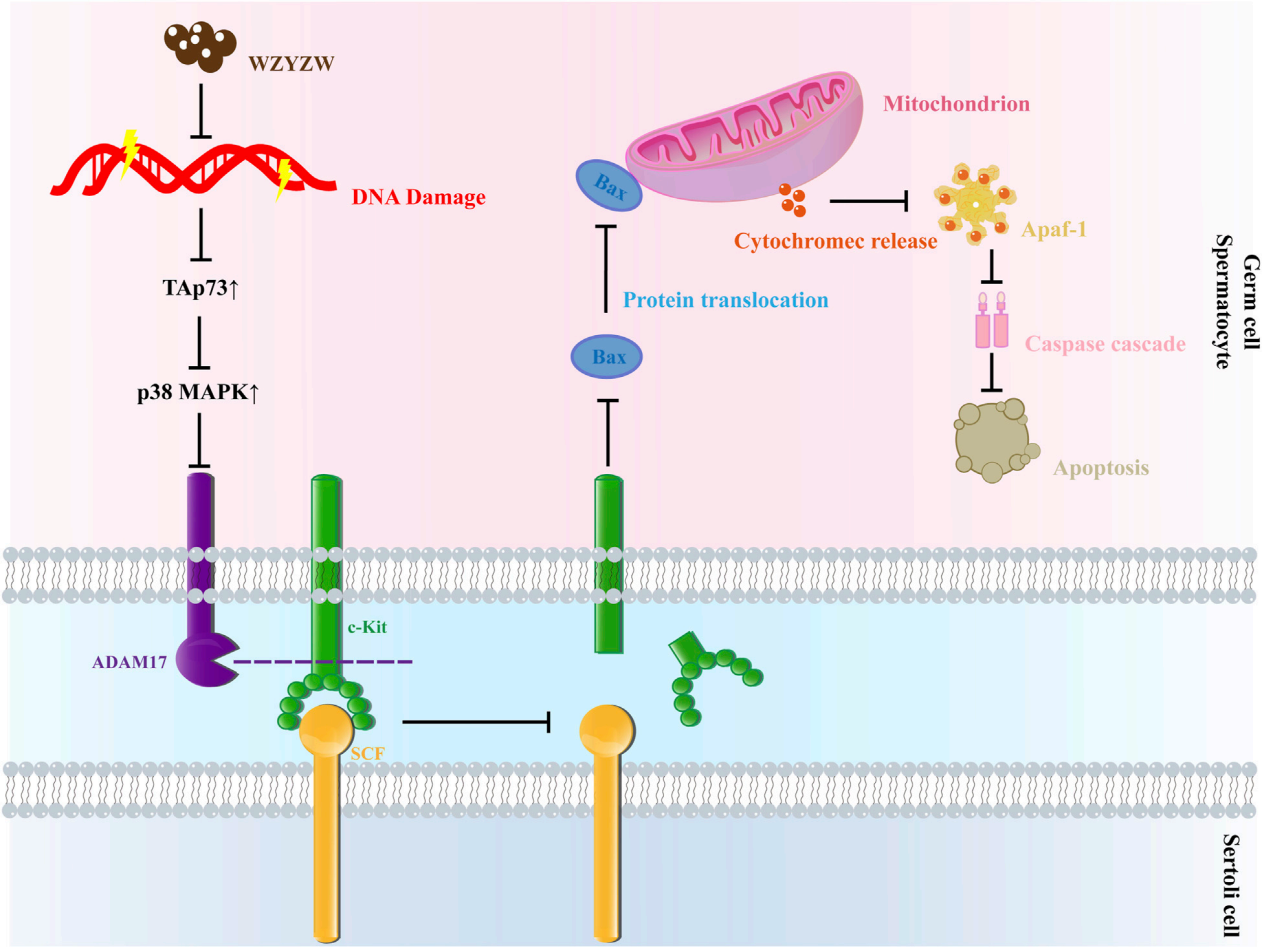
Schematic diagram of mechanism.

Our findings demonstrate that exposure of germ cells to TGs or etoposide induces DNA damage, upregulates TAp73 protein expression, and activates the P38 MAPK-ADAM17 pathway, ultimately triggering mitochondrial apoptosis and resulting in OA. WZYZW attenuates germ cell DNA damage, downregulates TAp73 expression, and inhibits the mitochondrial apoptotic pathway by suppressing P38 MAPK-ADAM17 signaling, thereby preventing OA pathogenesis (Figure 8). This study provides novel experimental evidence supporting WZYZW’s efficacy against male reproductive injury and proposes new research avenues for elucidating its therapeutic mechanisms in OA treatment. Furthermore, this study is limited by its relatively small sample size, which may have impacted the generality and reproducibility of the experimental findings. Although the data demonstrated consistent and promising trends, future work will involve increasing the sample size and incorporating gain-of-function experiments to enhance the rigor and comprehensiveness of the research.

## Data Availability

The original contributions presented in the study are included in the article/Supplementary Material, further inquiries can be directed to the corresponding authors.
