# Supplementary material for: Wuzi-Yanzong-Wan inhibits testicular mitochondrial apoptosis in rats by downregulating TAp73-Mediated P38 MAPK-ADAM17 pathway
Source: Front Pharmacol. 2025 Sep 8;16:1665356. doi: 10.3389/fphar.2025.1665356 (PMC12450939; doi:10.3389/fphar.2025.1665356)
Supplement: Supplementary file 1 [file Supplementaryfile1.docx]

| Compound | Retention time/min | Ionization method | Multi-Reaction Detection Scanning | Declustering Potential /V | Collision energy /eV |
| --- | --- | --- | --- | --- | --- |
| Betaine | 0.97 | ESI^+^ | 118.1→58.1 | 50 | 38 |
| Hyperoside | 6.57 | ESI^+^ | 483.2→185.1 | 40 | 33 |
| Schizandrin | 12.77 | ESI^+^ | 433.1→415.4 | 115 | 15 |
| Deoxyschizandrin | 15.41 | ESI^+^ | 417.5→316.1 | 190 | 33 |
| Geniposidic acid | 3.72 | ESI^-^ | 373.1→123.1 | -50 | -26 |
| Ellagic acid | 6.46 | ESI^-^ | 301.1→284.1 | -150 | -41 |
|  |  | ESI^-^ | 301.1→145 | -150 | -49 |
| Acteoside | 6.61 | ESI^-^ | 623.1→461.3 | -40 | -39 |
|  |  | ESI^-^ | 623.1→161.1 | -40 | -42 |
| Kaempferol 3-rutinoside | 7.06 | ESI^-^ | 593.2→285 | -100 | -41 |
| Quercetin | 10.14 | ESI^-^ | 301.1→151.1 | -100 | -30 |
|  |  | ESI^-^ | 301.1→178.9 | -100 | -25 |
| Kaempferol | 11.05 | ESI^-^ | 285.1→117.1 | -160 | -51 |
|  |  | ESI^-^ | 285.1→159 | -160 | -42 |

Supplement Tab1. The parameters of mass spectrametry of the 10 compounds of WZYZW
