# Supplementary material for: Wuzi-Yanzong-Wan inhibits testicular mitochondrial apoptosis in rats by downregulating TAp73-Mediated P38 MAPK-ADAM17 pathway
Source: Front Pharmacol. 2025 Sep 8;16:1665356. doi: 10.3389/fphar.2025.1665356 (PMC12450939; doi:10.3389/fphar.2025.1665356)
Supplement: Supplementary file 2 [file Supplementaryfile2.docx]

Supplemental Figure 1. The total ion chromatogram and extracted ion chromatogram of each compound in WZYZW samples

（A is the MRM chromatogram of betaine; B is the MRM chromatogram of hyperoside; C is the MRM chromatogram of schizandrin A; D is the MRM chromatogram of deoxyschizandrin A; E is the MRM chromatogram of geniposidic acid; F is the MRM chromatogram of ellagic acid; G is the MRM chromatogram of acteoside; H is the MRM chromatogram of kaempferol-3-O-rutinoside; I is the MRM chromatogram of quercetin; J is the MRM chromatogram of kaempferol; K is the TIC chromatogram of WZYZW samples）
